# Embodied cognition and L2 sentence comprehension: an eye-tracking study of motor representations

**DOI:** 10.3389/fnhum.2024.1410242

**Published:** 2024-09-17

**Authors:** Ruei-Fang Shiang, Chiou-Lan Chern, Hsueh-Chih Chen

**Affiliations:** ^1^Department of English, National Taiwan Normal University, Taipei City, Taiwan; ^2^Department of Educational Psychology and Counseling, Chinese Language and Technology Center, Social Emotional Education and Development Center, Institute for Research Excellence in Learning Sciences, National Taiwan Normal University, Taipei City, Taiwan

**Keywords:** embodied cognition, eye tracking, L2 reading comprehension, mental representation, action sentence, motor, perception, ESL

## Abstract

**Introduction:**

Evidence from neuroscience and behavioral research has indicated that language meaning is grounded in our motor–perceptual experiences of the world. However, the question of whether motor embodiment occurs at the sentence level in L2 (second language) comprehension has been raised. Furthermore, existing studies on motor embodiment in L2 have primarily focused on the lexical and phrasal levels, often providing conflicting and indeterminate results. Therefore, to address this gap, the present eye-tracking study aimed to explore the embodied mental representations formed during the reading comprehension of L2 action sentences. Specifically, it sought to identify the types of motor representations formed during L2 action sentence comprehension and the extent to which these representations are motor embodied.

**Methods:**

A total of 56 advanced L2 learners participated in a Sentence–Picture Verification Task, during which their response times (RTs) and eye movements were recorded. Each sentence–picture pair depicted an action that either matched or mismatched the action implied by the sentence. Data analysis focused on areas of interest around the body effectors.

**Results and discussion:**

RTs in the mismatch condition indicated an impeding effect. Furthermore, fixations on the body effector executing an action were longer in the mismatch condition, especially in late eye-movement measures.

## 1 Introduction

Amodal theories of language processing suggest that linguistic meaning is mentally represented by abstract symbols (Burgess and Lund, 1997; Griffiths et al., 2007). However, these theories cannot explain the perceptual experiences evoked when reading or hearing sentences (e.g., “a blue live lobster” vs. a “red cooked lobster”: Mimi looked at the lobster *in the cold water*/*on a hot grill*) (Glenberg et al., 2004; Kiefer and Pulvermüller, 2012). Similarly, analyses based on amodal propositional representations overlook the differences in actions described by sentences like “Joyce is playing piano/violin” (i.e., key-pressing vs. bowing). To address these shortcomings, researchers who investigated embodied cognition have suggested that language comprehension involves the mental activation and integration of motor and perceptual experiences related to the events described by linguistic symbols (Zwaan, 2004; Borghi, 2012; Shapiro, 2019).

Converging empirical evidence from neuroscience and behavioral research supports the theory that language meaning is grounded in our motor–perceptual experiences of the world, thus favoring that language comprehension is embodied (e.g., Kaschak et al., 2005; Tettamanti et al., 2005; Casteel, 2011; Moreno et al., 2013; De Koning et al., 2017; Kronrod and Ackerman, 2021). One line of empirical evidence for this embodied approach to language meaning is action-based (Glenberg and Kaschak, 2002), which emphasizes motor embodiment. At the lexical level, Hauk et al. (2004) demonstrated that L1 action verbs develop their motor representations somatotopically in the motor and premotor cortex. This has inspired studies in L2 embodied pedagogy studies, leading to the design of action-oriented classroom activities to facilitate L2 vocabulary learning (e.g., Ulbricht, 2020; García-Gámez et al., 2021), though not as much in L2 reading comprehension.

However, several L1 studies on embodied representations at the sentential level show that motor embodiment is context-dependent (e.g., Santana and De Vega, 2013; Schuil et al., 2013). A recent L2 study found that the motor representations of L2 action verbs are less or not grounded in motor experiences compared to L1 action verbs (Tian et al., 2020). To the best of the authors' knowledge, no L2 study of this kind has yet explored motor embodiment at the sentential level. Therefore, this study aimed to investigate which motor representations are constructed during the comprehension of L2 action sentences.

### 1.1 Mental representations and embodiment

To understand a text, individuals not only represent the text itself but also build mental representations, or situation models, of the scenarios described (Zwaan and Radvansky, 1998; Magliano et al., 1999). An embodied account of language comprehension argues that motor–perceptual representations are integral to constructing these mental representations (Barsalou, 1999; Zwaan, 1999). By activating and integrating motor–perceptual experiences, individuals can gather and elaborate on information about the events described in a text, leading to a high level of language comprehension (Zwaan, 2014; De Vega, 2015). That is, mental representations are multimodal, involving perceptual and motor representations, and are therefore embodied. Constructing mental representations can be challenging for less skilled language users, as it requires higher-order and deep-level processing (Zwaan and Taylor, 2006). Empirical evidence shows that L2 users often find tasks requiring such processing inefficient and effortful (Francis and Gutiérrez, 2012; Horiba, 1996; Pérez et al., 2019). Thus, building mental representations appears particularly challenging for L2 users. Notably, Zwaan and Brown (1996) found that English learners of French developed weak and reduced mental representations of French stories, making fewer inferences, while their mental representations of English stories were strong and comprehensive.

### 1.2 Embodiment in L1

The compatibility effect, frequently observed in studies using a Sentence–Picture Verification Task (SPVT), offers behavioral evidence that individuals mentally create motor–perceptual representations to comprehend sentences in L1. For example, in Zwaan and Pecher's (2012) research on perceptual representation, a facilitating effect (i.e., a shorter reaction time, RT) was observed when the shape/orientation of an object implied in a sentence matched what was seen in a picture. Specifically, RTs were shorter when participants saw an image of a bird with outstretched wings after reading “There is a bird in the sky.” Conversely, an impeding effect, characterized by a longer RT, was observed when there was a mismatch between the sentence and the picture, such as when the sentence was “There is a bird in the nest.” Similarly, Holt and Beilock (2006) employed an SPVT to show that native English speakers mentally construct motor representations when reading action sentences in L1. In summary, the facilitating and impeding effects observed in the SPVT indicate the formation of motor or perceptual representations. Ferstl et al. (2017) investigated whether manipulating action performers' facial appearances and clothing affected viewers' judgments of differences and similarities between two ambiguous actions. Their results showed that visual representations of actions, along with the facial identities of the action performers, influenced viewers' ability to distinguish between the actions. Notably, both Ferstl et al. (2017) and Holt and Beilock (2006) used picture stimuli depicting only the action performers' hands in motion (i.e., representing affordances) without showing the actual objects involved. This similarity provides useful guidelines for displaying picture stimuli in motor embodiment studies.

### 1.3 Motor embodiment in L1: action-based sentence comprehension

The Indexical Hypothesis (IH) is an embodied approach to language comprehension. It emphasizes situated action and suggests that the meaning of a situation is determined by the series of actions individuals take to interact with the physical world. Accordingly, sentence comprehension relies on bodily action and involves three processes: indexing, extracting and inferring affordances, and meshing (Glenberg and Robertson, 1999; Kaschak and Glenberg, 2000). To determine whether a sentence is meaningful, individuals index or map words/phrases to their physical referents at the beginning of the process of understanding the sentence. Subsequently, they derive potential affordances related to these references. Affordances represent how individuals interact with referents and their applications in various situations. For example, to move luggage, travelers pull the collapsible handle. Finally, the grammatical structure of the sentence guides the meshing of these affordances to execute the intended action. Here is an example of how this process works: “He hangs the jacket on the collapsible handle of upright luggage.” The syntactic structure of this sentence guides readers to integrate specific actions implied in the sentence to achieve the goal of hanging up the jacket, such as extending the collapsible handle from the wheeled bag of the luggage and draping the jacket over it. However, meaninglessness arises when affordances are meshed into impossible actions (e.g., “he hangs the jacket on the upright bowl”). The ability to successfully mesh affordances into a coherent set of actions depends on the relationship between the affordances of the individual (i.e., he), the objects involved (i.e., jacket, luggage/bowl), and the goal specified in the sentence context.

Santana and De Vega (2013) illustrated the action-based sentence comprehension model. Their study found an enhanced N400 effect when participants read sentences describing a protagonist performing two actions simultaneously (e.g., *while applying ointment to her wounded hand, she stretched a* ***bandage***). N400 is a brain signature sensitive to semantic or world knowledge inconsistencies. However, Santana and De Vega (2013) explained that the increased N400 observed in their study demonstrated a motor incongruency effect rather than a semantic violation, as their sentence stimuli did not contain semantic anomalies. Moreover, the two actions (i.e., stretching a bandage and applying ointment) were consistent with the scene described in the sentences, indicating that world knowledge violations did not contribute to the increased N400. This explanation was further supported by another experiment from the same study, which found that the N400 effect diminished when the two actions were performed consecutively. They explained further that the observed N400 reflected the unsuccessful formation of motor representations in the brain's motor regions due to a failure in the affordance meshing of the two actions. They also noted that the N400 effect peaked at the end of the sentence (i.e., bandage), not at the mention of the second verb (i.e., stretched). Based on these findings, Santana and de Vega suggested that motor representations are formed when sufficient contextual information is provided. This suggestion appears to conflict with the theory that action verbs alone activate motor embodiment (Hauk et al., 2004). However, Schuil et al. (2013) found that the motor region was activated when action verbs were embedded in literal sentences (e.g., Peter picks up the books after the examination) but less activated in nonliteral sentences (e.g., Peter picks up the pieces after the examination). This highlights the essential role of sentence context in activating and integrating motor experiential traces for developing motor representations.

### 1.4 Embodied aspects of mental representations revealed through eye-tracking data

Oculomotor recording has proven to be a valuable tool for uncovering the mental representations formed for sentence comprehension (Anderson and Spivey, 2009) and has been employed to explore embodied language comprehension. For example, dwell time and fixation counts on the depicted protagonist and destination were shorter and fewer for sentences describing fast actions compared to those describing slow actions (e.g., *A man dashed/sauntered into the supermarket*). In other words, the speed of a motion described in a sentence influenced the amount of gaze directed at the protagonist (i.e., the man) and the destination (i.e., the supermarket) relevant to the motion (Lindsay et al., 2013; Speed and Vigliocco, 2014). The changes in dwell time and fixation counts on the depicted protagonist and the destination indicate that indexing occurs in two directions. One approach maps words or phrases to their mental/internal representations (Glenberg and Robertson, 2000), while the other links external/physical elements to these mental/internal representations (Spivey and Richardson, 2009). The eye-tracking studies above showed that embodied indexing occurs in two directions simultaneously. Specifically, verbal inputs create mental representations, which are then linked/indexed to the externally and pictorially presented protagonist, destination, and event.

### 1.5 Embodiment in L2

Pavlenko (2014) argued that L2 is disembodied because bilinguals are generally exposed to L2 in grammar-based, amodal symbol-oriented instructional contexts. Using an SPVT, Norman and Peleg (2022) found that Hebrew L2 English readers did not show signs of constructing perceptual representations when comprehending English. Similarly, Chen et al. (2020), employing a delayed SPVT, found no perceptual embodiment in L2 Chinese and L3 English. Conversely, Vukovic and Williams (2014) showed that perceptual, experiential traces were automatically activated when English learners of Dutch processed English sentences describing perceptual information during an SPVT. Similarly, Ahn and Jiang (2018), using an SPVT, found that late L2 Korean learners developed perceptual representations for Korean sentences related to shape or orientation. Of particular interest is Foroni (2015) study, which observed that Dutch L2 English learners' cheek muscles for smiling were stimulated when reading sentences expressing affirmative emotions (e.g., I am grinning). This finding was consistent with his previous L1 study of this kind. However, unlike L1 users in his previous study, L2 readers did not show inhibited cheek muscle activity when reading sentences expressing negative emotions (e.g., I am not grinning). Therefore, Foroni (2015) concluded that emotional language comprehension in L2 is less embodied than in L1 (see Zhang and Vanek, 2021 for a discussion of the processing cost of negation in L2). Regarding motor representations in L2 processing, Vukovic and Shtyrov (2014) observed that the motor experiential traces activated by action verbs were weaker for L2 (English) than for L1 (German). However, Tian et al. (2020) found the opposite. In their study, three types of English and Chinese verb phrases were produced as stimuli, with each conveying a different meaning: abstract, literal, and metaphorical. They found that motor activation strength increased from abstract to metaphorical to literal verb phrases in both languages. However, the motor activation for verb phrases was stronger in L2 (English) than in L1 (Chinese). They concluded that the strong motor responses observed in L2 verb phrases indicated that participants were processing less automatic language rather than solely reflecting motor representations. Therefore, they supported the view that L2 action-related language is processed in a disembodied manner.

### 1.6 The present study

Research on the role of perceptual traces in L2 comprehension has yielded mixed results. While previous L2 studies on perceptual embodiment have focused on lexical and sentential levels, studies on motor embodiment in L2 have been limited to the lexical and phrasal levels, often reporting conflicting and indeterminate results. However, L1 studies (Santana and De Vega, 2013; Schuil et al., 2013) have suggested that sentence context is crucial for activating and integrating motor experiential traces. Moreover, these traces are essential for forming motor representations needed to comprehend action sentences. Therefore, it is important to explore whether motor representations are developed at the sentential level in L2, consequently motivating this study to explore the motor aspects of mental representations (i.e., motor representations) formed for L2 action sentence comprehension. This study addresses the following research questions (RQs):

RQ 1: What embodied aspects of mental representations are involved in L2 action sentence comprehension? Specifically, what motor experiential traces contribute to these mental representations?
RQ 2: To what extent are these L2 mental representations grounded in motor experiential traces?

Using the SPVT paradigm and the eye-tracking technique, this study compared L2 readers' eye gaze while viewing matched and mismatched sentence-picture pairs. Specifically, the picture depicted a protagonist performing an action that either corresponded with or differed from the action implied in the sentence. Readers' eye gaze was recorded as they assessed whether the picture matched their comprehension of the sentence stimuli. As mentioned in Section 1.4, fixations index the development of mental representations. Therefore, analyzing participants' eye gaze on the picture will reveal the motor representations they form to comprehend the sentence.

This study operationalized the formation of motor representations using IH. Specifically, the answers to RQ 1 were derived from analyzing how L2 participants gaze at and examine the action and the protagonist's body effectors (i.e., head, hands, and legs) in the picture stimuli. Because action performers' identity (i.e., facial appearance/facial identity) affects observers' ability to correctly identity the actions of the action performers (Ferstl et al., 2017), our first hypothesis (HP) is as follows:

HP1
*If L2 participants form motor representations for comprehending an action sentence stimulus, their eye gaze will focus on the protagonist's head in the picture stimulus*.

Specifically, participants were expected to index/map the protagonist's head in the picture to the protagonist described in the action sentence. That is, they would index the visual representation of the protagonist to the mental referent of the protagonist formed for comprehending the action sentence. This indexing would allow participants to determine if the depicted protagonist matched the one described in the sentence.

We also examined the relationships between the depicted body effectors, the implied action, and the described object based on the reference status in the sentence of the body effectors. The following hypotheses address affordances and meshing.

HP2
*If the affordances of the protagonist and the described object are successfully meshed, the eye gaze on the picture will shift to the body effector performing the action implied in the sentence. Moreover, the number of gazes on this body effector will be higher in the mismatch condition than in the match condition*.
HP3
*When motor representations are developed, both the match–mismatch conditions of the sentences and the reference status of the body effectors in the sentence will jointly influence participants' eye gaze on the picture stimuli*.

In summary, RQ 1 can be answered by examining which parts of the picture stimulus participants focus on and inspect and how frequently they do so. Although this study primarily focused on motor representations of hand actions, the picture stimuli also included the protagonist's legs. It was anticipated that the legs, compared with the hands, would have weaker relationships with the hand action and the described object. Consequently, participants were expected to focus more on the hands than on the legs. When the leg was presented in the picture stimuli, it was used as a distractor to assess whether the participants correctly formed the motor representation of hand actions. Previous studies employing the SPVT paradigm suggest that the facilitating and impeding effects reflect that motor–perceptual representations were formed to comprehend the sentence stimuli before the picture stimuli were displayed (Holt and Beilock, 2006). Therefore, we expected to see the two effects in this study.

Additionally, this study examined the extent to which the L2 mental representations are experientially embodied in motor terms, particularly focusing on the timing effect (i.e., early/late eye movements). Specifically, it investigated how quickly or slowly L2 participants identified actions depicted in picture stimuli that either matched or mismatched the actions described in the sentence stimuli. L2 mental representations tend to be reduced and weaker because of the slow and demanding nature of higher-order and deep-level processing for L2 users (Horiba, 1996; Zwaan and Brown, 1996). Based on previous findings, we propose the following hypothesis:

HP4:
*If L2 motor representations are weak and lack sufficient motor experiential traces, identifying whether the action depicted in the picture matches or mismatches the action described in the sentence will be slow and delayed. Conversely, stronger L2 motor representations will lead to quicker identification*.

## 2 Materials and methods

### 2.1 Participants

Overall, 56 right-handed L1 Chinese-speaking students (21 men, 35 women; mean age = 20.4 years, *SD* = 1.1) with normal or corrected-to-normal vision were recruited from universities in northern Taiwan. To participate, they were required to have taken a reading test from a globally recognized English proficiency test (e.g., TOEFL or IELTS) within the past 2 years and provide a copy of their test score report.

All participants had English reading abilities at the C1 level on the CEFR scale^1^. We initially recruited 79 students for the experiment, but 23 of them were excluded from further analyses due to the following reasons: minoring in English, high rates of incorrect responses on verification tasks and comprehension tests, invalid RTs, invalid fixation durations, and technical issues during data collection. Details on data cleaning and analysis are provided in Section 2.7. The remaining 56 participants were majoring in engineering or medicine.

G^*^Power software Version 3.1.9.7 (Faul et al., 2007) was used to conduct a priori power analysis to estimate the optimal sample size for the 3 (body effectors) × 2 (match–mismatch conditions) repeated measure design. A large effect size (*f*^2^) of 0.35 was used, with an alpha of 0.05, a power of 0.80, and five predictor variables. The results showed that a minimum sample size of 43 was needed to detect a large effect size with an actual power of 0.81. Therefore, our final sample size (*N* = 56) was adequate. Superpower 0.20 (Lakens and Caldwell, 2021) was used to conduct a simulation-based priori power analysis. We specified a sample size of 20 per cell, a common *SD* of 1, a common correlation of 0.70, and means of 1, 1.3, 1, 2.05, 0, and 0.1 for the six cells, respectively, with an alpha of 0.05. The results showed that this study would achieve an expected power of 0.90 with an effect size of *f*^2^ = 0.34. Thus, our final sample size of 28 per cell was adequate.

### 2.2 Experimental design

This study employed the SPVT combined with eye-tracking techniques to investigate how the reference status of body effectors and the match–mismatch conditions in sentences influenced participants' eye gaze on the three body effectors depicted in the picture stimuli. The study featured two repeated measures as independent variables: body effectors and match–mismatch conditions. For body effectors, there were three levels: head, hand, and leg. Each body effector was categorized according to its reference status within the sentence, reflecting the relationships among the three body effectors, the implied action, and the described objects. For example, in the sentence “Mr. Bean is measuring the length of cars in a car park,” the name “Mr. Bean” explicitly identifies the protagonist and, by extension, the Head body effector. The Hand body effector, however, is implicitly involved, as the action “measuring” suggests the use of hands, though it is not directly mentioned in the sentence. The leg body effector, neither explicitly nor implicitly referenced, serves as a distractor. Therefore, the body effectors were categorized as follows:

Mentioned (Head, Hand) vs. Not-mentioned (Leg)
Explicitly mentioned (Head) vs. Implicitly mentioned (Hand)

For match–mismatch conditions, we manipulated whether the action implied in the sentence matched or mismatched the action depicted in the picture (i.e., action-matching sentence vs. action-mismatching sentence).

To answer RQ 1, a 3 (Body effectors: Head, Hand, Leg) × 2 (Conditions: Match, Mismatch) repeated measure design was applied to eye movement data: fixation counts^2^. The independent variables were body effectors and match-mismatch conditions, with fixation counts as the dependent variable. Moreover, two 2 × 2 repeated measure designs were used for further analysis:

(A) 2 (Mentioned: Head and Hand vs. Not-mentioned: Leg) × 2 (Match vs. Mismatch Conditions).
(B) 2 (Explicitly mentioned: Head vs. Implicitly mentioned: Hand) × 2 (Match vs. Mismatch conditions).

Comprised two repeated measures of independent variables: mentioned vs. not-mentioned body effectors and match vs. mismatch conditions, each with two levels. Analysis of (A) determined whether and how these variables influenced participants' fixation counts on the body effectors depicted in the picture stimuli. Similarly, (B) involved two repeated measures of independent variables, each with two levels. Analysis of (B) assessed how these variables influenced participants' fixation counts on the body effectors depicted in the picture stimuli. To address RQ 2, a one-way repeated measures design was used, with match–mismatch conditions serving as the independent variable and the eye movement data for temporal measures^3^ in the Hand region serving as the dependent variable.

### 2.3 Materials

**Figure 1:**
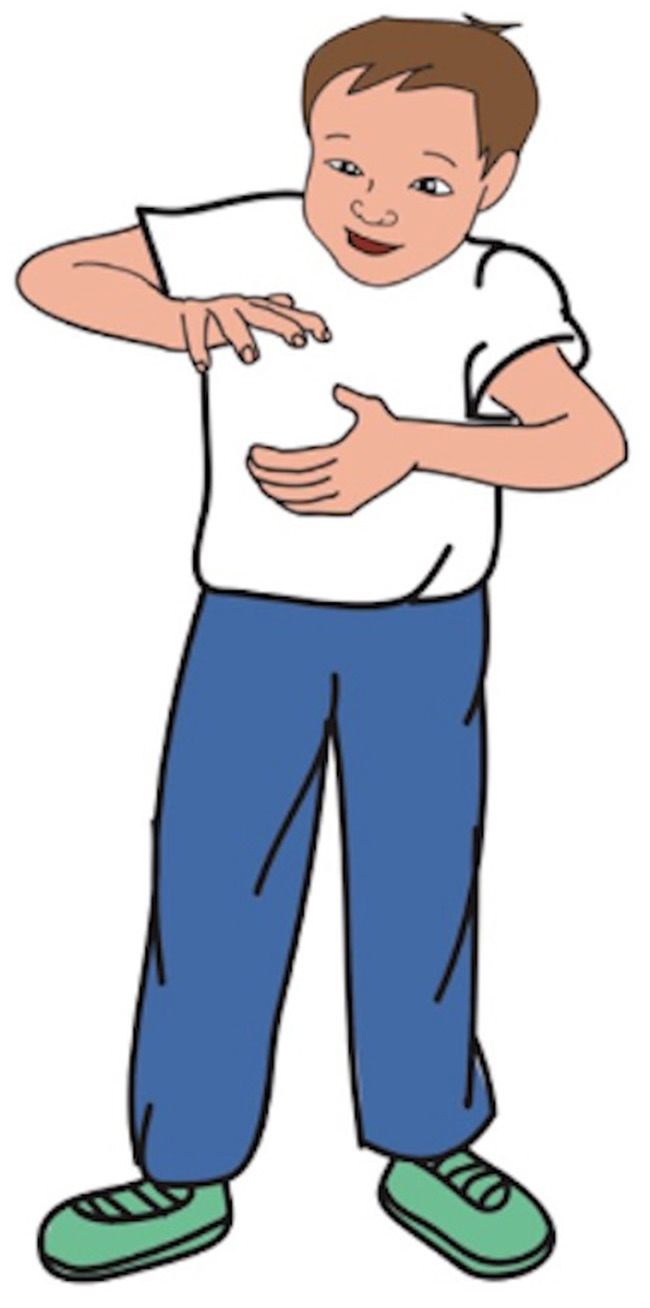
Picture in sample 1.

A total of 24 critical sentence–picture item sets were created, each consisting of two sentences and one full-color picture drawn by a professional artist^4^. Each of the two sentences implied a different action (i.e., each item set had two implied actions). Both sentences contained the same verb, and the implied actions were performed by the same body effector of the same protagonist. However, the picture in each item set matched the action implied by only one of the sentences (i.e., action-matching sentence vs. action-mismatching sentence; see Table 1, Figure 1 for examples). During each trial, participants were shown only one sentence from a given critical sentence–picture item set.

**Table 1:** Some samples of SPVT pairs with comprehension test items.

**Sentence-picture pairs of a critical trial: sample 1**	
*The sentences in Sample 1 Mismatch* A boy is opening a can of Coke in the metro car. *Match* A boy is opening a jar of jam in the metro car. *Comprehension test item*: A boy is opening a carton of milk in the metro car.	Figure 1. The picture in sample 1. 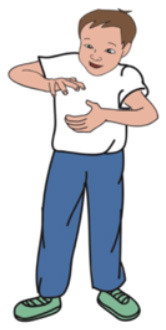
**Sentence-picture pairs of a critical trial: sample 2**	
*The Sentences in Sample 2 Match* A grandma is filling teacups with green tea in the dining room. *Mismatch* A grandma is filling cupcakes with vanilla cream in the dining room. *Comprehension test item*: The grandma is filling a pie with minced beef in the dining room.	Figure 4. The picture in sample 2. 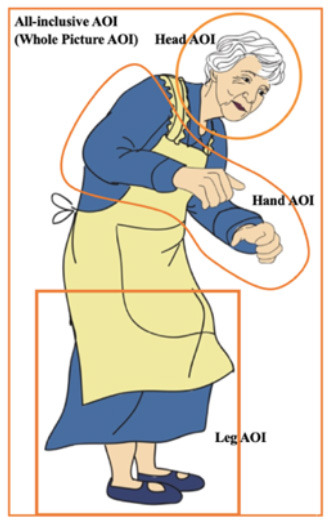
**A sample of filler pair**	
*The sentence in the sample of filler pair* A director is filming a hyena wrestling with its partner for food. *Comprehension test item*: A director is filming a hyena wrestling with its partner for territory.	Figure 2. The picture in the sample of the filler pair. 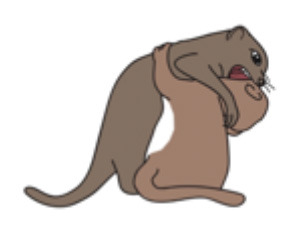

The verbs were selected based on their definitions in the Cambridge and Oxford English Dictionaries, with the criterion that neither dictionary could explicitly indicate which body effector executes the action described by the verb. For example, the verb “catch” was excluded because its definition, “to stop and hold a moving object or person, especially in your hands,” implies the use of hands.

**Figure 2:**
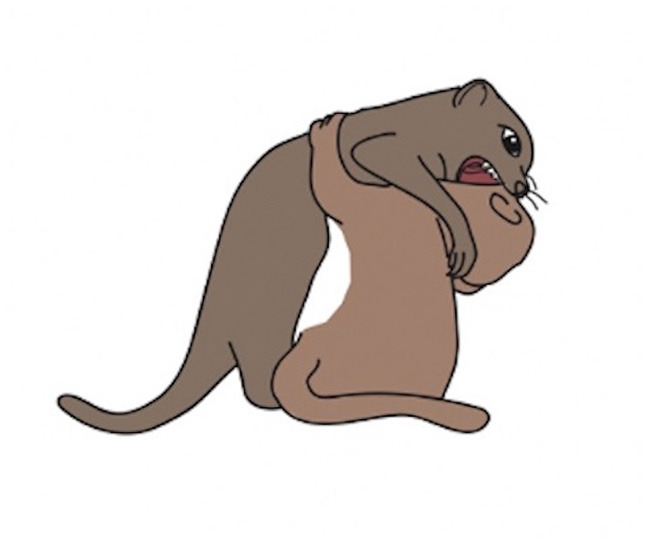
Picture in the sample of filler pair.

This study focused on making inferences about hand- and arm-related actions. While the way an action is performed and the body effector associated with the action were not explicitly stated, they could be inferred from the context. Each sentence presented to the participants explicitly mentioned a protagonist. Sentence length and the protagonist's position in the sentence were consistent across sentence stimuli in both the match and mismatch conditions. In summary, although both sentences in any given sentence–picture item set contained the same verb, the action depicted in the picture matched the action in only one of the sentences. Notably, the specific details of the action were not explicitly mentioned but needed to be inferred from the sentence context. Additionally, filler pairs were included to prevent participants from discerning the purpose of the study. These filler sentences described objects, locations, or general situations and were paired with pictures that were similar but irrelevant to the meaning of the sentences (Table 1, Figure 2). Moreover, yes/no comprehension tests were developed for critical and filler items to verify that participants understood the sentences used in the verification tasks. For instance, the comprehension test for the “boy” sentence–picture item set shown in Table 1 is: “A boy is opening a carton of milk in the metro car.”

A separate group of 10 advanced L2 readers participated in a pilot study. The recruitment criteria of these participants were similar to those for the main study. The pilot study aimed to confirm that none of the words in the sentences—whether critical items or fillers—were unfamiliar to the participants and that the correct action could be inferred from each sentence stimulus. In the pilot study, participants rated the compatibility of each sentence stimulus with the corresponding illustrated action on a 7-point Likert scale, ranging from “very compatible” to “very incompatible.” Sentence–picture pair rated below 5 was excluded. Additionally, items that consistently induced prolonged RTs or unusual eye gaze^5^ were excluded. Ultimately, 12 critical item sets and 24 fillers were retained for the experiment. The average compatibility rating of the critical items in the match condition was 6.5 (*SD* = 0.60).

In summary, two lists of SPVT item sets (List 1 and List 2) were created. Both lists contained the same filler items. The critical items and sentence conditions (match and mismatch) were counterbalanced across the two lists. This means that if a critical item set presented its match condition on one list, it showed its mismatch condition on the other. Specifically, each picture in a critical item set appeared once in each list, paired with the action-matching sentence on one list and the action-mismatching sentence on the other. Moreover, each list comprised one practice block and three experimental blocks, with an equal number of filler pairs and matching and mismatching pairs in each block. Within each block, the critical pairs and filler pairs were randomly displayed. The order of the three experimental blocks was also randomized. Comprehension tests were administered after each block. Participants were randomly assigned to one of the two lists: half viewed List 1, while the other half viewed List 2.

### 2.4 Procedure

Participants began with practice trials to familiarize themselves with the task. A 9-point calibration was used to adjust the eye-tracking apparatus. During the experiment, drift correction was performed before each sentence–picture pair presentation. Each sentence was displayed on the screen one at a time, centered and left-justified. A black cross was displayed for 100 ms before each sentence stimulus to direct participants' attention. The black cross was positioned to the left of the center of the screen, on the first letter of each sentence. Once participants had read and understood the sentence, they pressed the space bar. A black cross then appeared in the center of the screen for 100 ms, followed by a blank screen for 250 ms. A picture stimulus was then displayed either to the left or right of the center of the screen. The second black cross was intended to focus participants' visual attention before presenting the picture stimuli.

**Figure 3:**
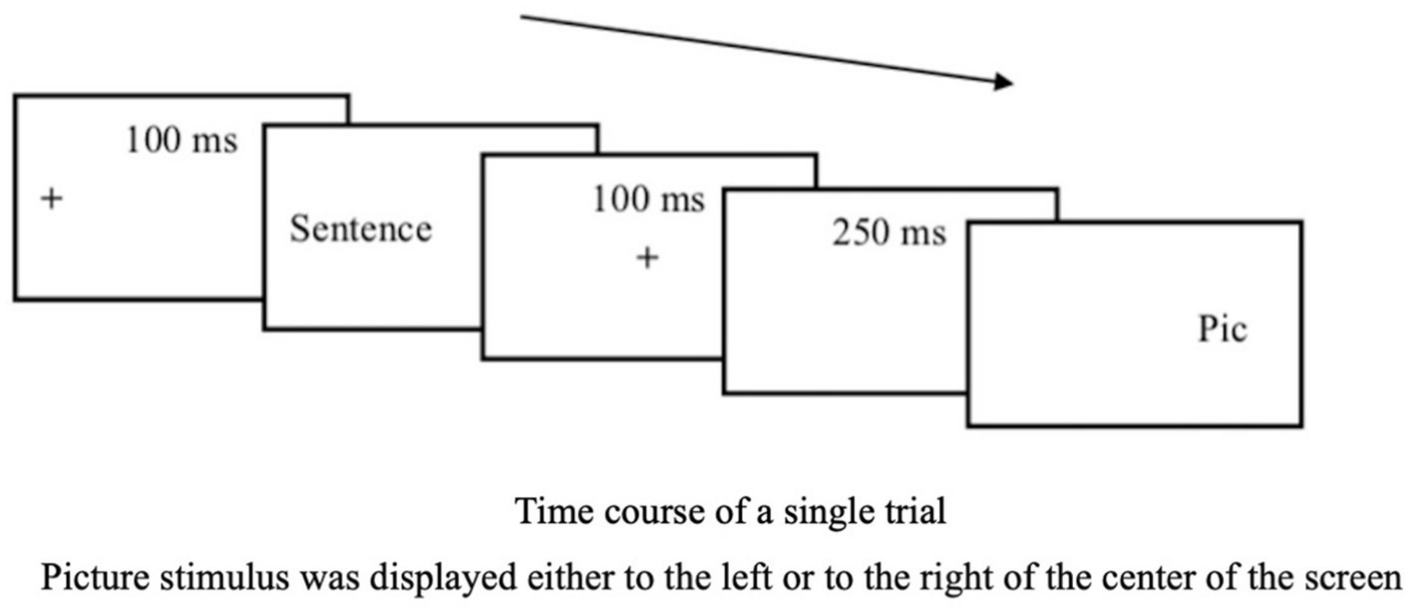
SPVT flowchart.

Participants were instructed to quickly determine whether the picture depicted any elements (e.g., a protagonist, place, object) mentioned in the preceding sentence, and their RTs were recorded. They used the computer keyboard to respond, pressing the “P” key labeled “Yes” if they believed the image was mentioned in the sentence and the “Q” key for “No” if they did not. Participants were not explicitly required to determine whether or not the action depicted in the picture matched the action implied in the sentence. Instead, they were simply asked to respond to “whether any element in the picture had been mentioned in the immediately preceding sentence.” The expected response for all of the critical trials was “yes,” while a “no” response was expected for all the fillers. As noted in Section 2.3, participants were randomly assigned to one of the two SPVT item set lists. Each list included one practice block and three experimental blocks. The comprehension test items were presented at the end of each block. Figure 3 illustrates the SPVT flowchart.

### 2.5 Apparatus

The EyeLink 1000 Plus eye-tracking system (SR Research Ltd.) with a 1,000 Hz sampling rate was used to record eye movements. Only the dominant eye was monitored, although viewing was binocular. Pictures and sentences were displayed on a 21-inch LCD monitor with a 1,440 × 900 screen resolution and a 50 Hz refresh rate. Participants sat 69 cm from the screen, with their heads stabilized by a chin-and-forehead rest to minimize head movement. The picture stimuli subtended a visual angle of 33.27° × 39.85° (width × height). Finally, sentences were displayed in 24-point Courier New font, black on a white background.

### 2.6 Measurements

This study used a standard SPVT approach, measuring the RT required for participants to determine if anything presented in the picture was mentioned in the immediately preceding sentence. This method helped avoid inadvertently revealing the research objective to the participants during the task. Only RTs for the critical items were included in the statistical analysis. Additionally, eye movement data were analyzed to assess the processing time as participants visually attended to predetermined areas of interest (AOIs) in the picture stimuli.

**Figure 4:**
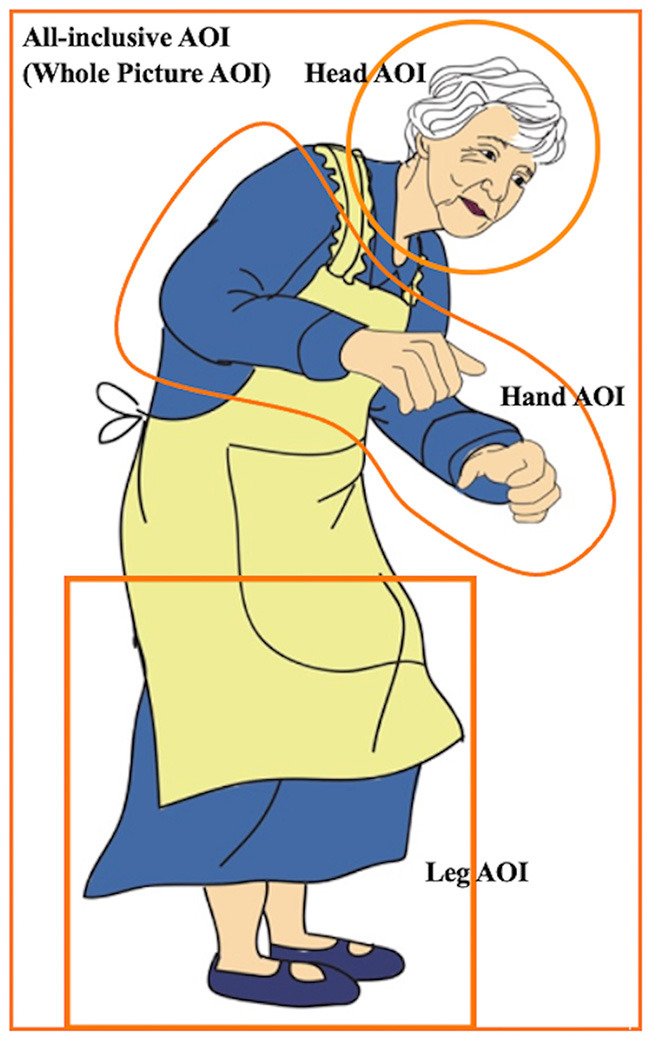
Picture in sample 2.

The measurements are detailed in Table 2. Each body effector (the Hands, Head, and Legs) was assigned a specific AOI. Additionally, an All-Inclusive AOI covered the protagonist in the image (the whole picture) (Figure 4). The four AOIs, shown in the picture of the older woman in Table 1, enabled both general and specific inferences of the data. Each of the six eye movement measures detailed in Table 2 corresponds to a different time event (Godfroid, 2019). The Hand AOI was used to assess data on indicators (a)–(f), the All-Inclusive AOI was used for (e) and (f), and the Head and Leg AOIs for (f). This study focused exclusively on motor representations of hand- and arm-related actions. Therefore, the results for RQ 2, indicated by temporal eye movement measures, reflect the degree of motor embodiment of the Hand action developed by the participants. Conversely, RQ 1 results, shown by fixation counts, revealed which parts of the picture participants examined. Overall, RQ 1 was addressed through fixation counts (f) in the Head, Hand, and Leg AOIs, while RQ 2 was answered through temporal measures (a)–(e) in the Hand AOI.

**Table 2:** Eye movement measures adopted in this study.

**Measure**	**Definition**	**Indicator of Timing Effects**
(a) First fixation duration	The time spent fixating on an AOI for the first time, reflects the processes of identification and recognition (De Graef et al., 1990)	Initial processing time (i.e., early eye movement measures)
(b) First-pass dwell time	The sum of the duration of all fixations falling within an AOI before moving to another AOI, which reflects the process of object recognition and tends to increase when an unexpected object is noticed (Henderson et al., 1999)	Early
(c) Second-pass dwell time	The time spent on the previously fixated AOI, excluding the first-pass dwell time, suggests that the participant engaged in reanalysis and intentional processing of the AOI	Late processing time (i.e., late eye movement measures)
(d) Regression-in-count	The number of times that an AOI, which is currently being viewed after having been processed previously, is returned to while looking at other AOIs, or in other words, the number of revisits	Late
(e) Total dwell time	The sum of all the dwell times for the same AOI during a single trial is an indication of the overall cognitive effort made while processing stimuli	Late
(f) Fixation count (i.e., the number of fixations falling within an AOI)	The strength of the participants' attentiveness to the AOI	Global indicator

The accuracy of the responses to the reading comprehension tests and the verification task were recorded. The reading comprehension test was designed to elicit a simple yes/no response (Table 1). The test results reflected the participants' attention to and understanding of each sentence stimulus. The comprehension tests were included based on the idea that people form comprehensive mental representations of what they read only when they read for understanding or learning (Zwaan, 2014). Accordingly, data were removed if the accuracy of either the reading comprehension tests or the SPVT was lower than 80%^6^.

### 2.7 Statistical analyses and data cleaning

This study used a repeated measures design. All data analyses were conducted using R software, Version 3.6.1 (R Core Team, 2019). Criteria for valid fixation and RT were strictly enforced for data cleaning. If any fixation or RT from a participant did not meet these criteria, all data collected from that participant were removed from the analyses^7^. The data are accessible at this website: https://osf.io/zeufn/s.

#### 2.7.1 Behavioral data cleaning and analysis

The average accuracy rates for the verification task and comprehension test were 96.3% and 87%, respectively. Only RTs within three *SDs* of a participant's mean RT (mean ± 3 *SD*) for any single trial were considered valid. To determine whether impeding and facilitating effects occurred, a pairwise t-test was performed on the RTs.

#### 2.7.2 Eye-tracking data cleaning and analysis

The temporal threshold for identifying valid ocular fixation was set at 50 ms^8^. Despite strict data cleaning, the second-pass dwell time data remained asymmetrically distributed. This issue was resolved by applying a square-root transformation, resulting in a better approximation of a normal distribution.

To answer RQ 1, we first computed a pairwise *t*-test on total dwell time and fixation counts to determine if global eye movement measures reflect impeding and facilitating effects related to motor embodiment. We then employed a 3 (Body effectors: Head, Hand, Leg) × 2 (Conditions: Match, Mismatch) repeated measures design to test the hypotheses of RQ 1. Data analyses were conducted on fixation counts using a linear mixed-effects model with the *lme* function in the *nlme* package, Version 3.1-141 (Pinheiro et al., 2021). To analyze the main effects and interactions, three orthogonal contrasts were set. Contrast 1 compared the match-to-mismatch condition of the sentences. Contrasts 2 and 3 compared the relationships among the three body effectors, the implied action, and the described object (the reference status in the sentence). Specifically, Contrasts 2 and 3 explored whether the participants' inspection of the picture stimuli was impacted by the mention of the body effector and whether it was explicit or implicit. In summary, Contrast 1 compared match vs. mismatch conditions, Contrast 2 compared mentioned vs. not-mentioned body effectors, and Contrast 3 compared explicitly vs. implicitly mentioned body effectors. Moreover, four models were built to identify the best fit for the fixation count data. The commands to run the four models in R are accessible at https://osf.io/zeufn/s. The specifications of the four models are as follows:

*The baseline model* included only an intercept, predicting the outcome solely from the intercept. The random part of this model showed that the two repeated-measures predictors, body effectors, and match-mismatch conditions, were nested within the participant variable. Moreover, maximum likelihood estimation was used for this model. Next, one predictor was added at a time. Keeping the same outcomes and predictors as *the baseline model*, we added body effectors to *the baseline model* as a predictor using the *update()* function to build *the body effector model*. Then, match–mismatch conditions were added to *the body effectors model* to create *the match–mismatch conditions model*. Finally, the interaction between match–mismatch conditions and body effectors was added to the *match–mismatch conditions model* to create *the interaction model*.

Every model was estimated using maximum likelihood, considering the random effects of the participants. The four models were compared using the *anova()* function. *Post-hoc* tests for the simple main effect of mentioned vs. not-mentioned body effectors, match vs. mismatch conditions, and explicitly vs. implicitly mentioned body effectors were conducted using pairwise *t*-tests with Bonferroni correction. Notably, the mentioned body effectors included fixation counts on Head AOI and Hand AOI. The simple main effect of the match vs. mismatch conditions in the 2 (mentioned vs. not-mentioned) × 2 (match vs. mismatch conditions) interaction was computed to compare the fixation counts on the not-mentioned Leg with the averaged fixation counts on the mentioned Head and Hand [(Head + Hand)/2 vs. Leg]. Afterward, a simple main effect of body effectors was assessed using the *glht* function in the *multcomp* package, Version 1.4-10 (Hothorn et al., 2008). Finally, paired *t*-tests with Bonferroni correction were performed on the selected eye-movement temporal measures to answer RQ 2.

## 3 Results

### 3.1 RTs during SPVT: facilitating and impeding effects were observed

The pairwise t-test was conducted to determine whether match-mismatch conditions affected RTs, which required participants to judge compatibility after viewing the picture stimulus. On average, RTs in the mismatch condition (*M* = 2,179.91, *SD* = 1,021.51) were significantly longer than in the match condition (*M* = 1,360.08, *SD* = 528.46, *t*(335) = −12.98, *p* < 0.001, *d* = 0.98, 95% CI [−944.04, −695.62]). This finding indicates a facilitating effect in the match condition and an impeding effect in the mismatch condition.

### 3.2 Whole-picture AOI: eye movement data reflect facilitating and impeding effects

When the entire picture was set as a single AOI (whole-picture AOI), the pairwise *t*-test showed that this region received more fixations in the mismatch condition (*M* = 5.02, *SD* = 2.02; *M* = 3.82, *SD* = 1.74) than in the match condition (*M* = 3.82, *SD* = 1.74; *t*(335) = −9.33, *p* < 0.001, *d* = 0.60, 95% CI [−1.08, −1.67]). Similarly, the paired samples *t*-test also demonstrated that total viewing time was significantly longer in the mismatch condition (*M* = 1,802.92, *SD* = 981.08) than in the match condition (*M* = 1,061.07, *SD* = 528.18; *t*(335) = −12.13, *p* < 0.001, *d* = 0.90, 95% CI [−862.10, −621.59]). This indicates an impeding effect in the mismatch condition.

### 3.3 Results for RQ 1: what embodied aspects of mental representations are formed to achieve L2 action sentence comprehension? Specifically, what motor experiential traces are involved in these mental representations? Three (Body effectors: Head, Hand, Leg) × Two (Conditions: Match, Mismatch) repeated measure design

**Figure 5:**
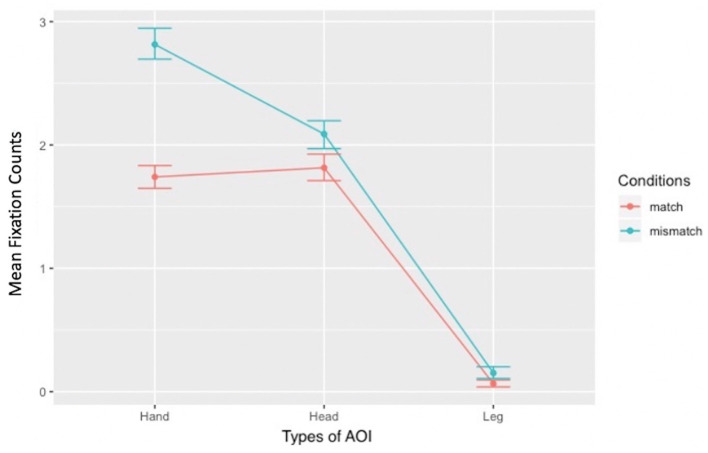
Main interaction effect.

RQ 1 explored which parts of the picture stimulus participants looked at and inspected, and how often they looked at those parts. Fixation count data collected for RQ 1 were analyzed using a linear mixed model approach. The contrasts built for the analyses were match vs. mismatch conditions, mentioned vs. not-mentioned body effectors, and explicitly vs. implicitly mentioned body effectors. Four models were also constructed: *the baseline model, body effectors model, match-mismatch conditions model, and interaction model* (see Sections 2.2 and 2.7 for details on contrasts and models). To identify the best-fitting model, the four models were compared using the *anova()* function. The results showed that *the body effectors model* fit the data better than *the baseline model* [χ^2^(2) = 482.81, *p* < 0.001], and *the match–mismatch conditions model* fit better than *the body effectors model* [χ^2^(1) = 53.45, *p* < 0.001]. However, the best fit was the *interaction model* [χ^2^(2) = 79.05, *p* < 0.001], as confirmed by the Akaike Information Criterion: *the baseline model* = 5,747, *the body effectors model* = 5,268, *the match–mismatch conditions model* = 5,217, and *the interaction model* = 5,142. This indicates that the fixation counts were significantly affected by the match-mismatch conditions and the type of body effectors (Match: Hand: *M* = 1.74, *SD* = 0.89; Head: *M* = 1.81, *SD* = 0.97; Leg: *M* = 0.06, *SD* = 0.27; Mismatch: Hand: *M* = 2.82, *SD* = 1.19; Head: *M* = 2.09, *SD* = 1.07; Leg: *M* = 0.15, *SD* = 0.44). Figure 5 shows the interaction graph, illustrating the inspection pattern concerning fixation counts across the three body effectors, focusing on the non-parallel lines. The line representing the match condition illustrates that fixation counts were slightly higher on the Head than on the Hand. However, the line representing the mismatch condition showed a different trend: fixation counts on the Hand were higher than on the Head. This match and mismatch conditions seemed not to affect the Leg. The higher position of the mismatch condition compared to the match condition line indicated that the mismatch condition resulted in higher fixation counts on the Hand and the Head. Further analyses of the interaction are provided in the following sections.

#### 3.3.1 Two (Mentioned: Head and Hand vs. Not-mentioned: Leg) × Two (Match vs. Mismatch conditions) interaction

**Figure 6:**
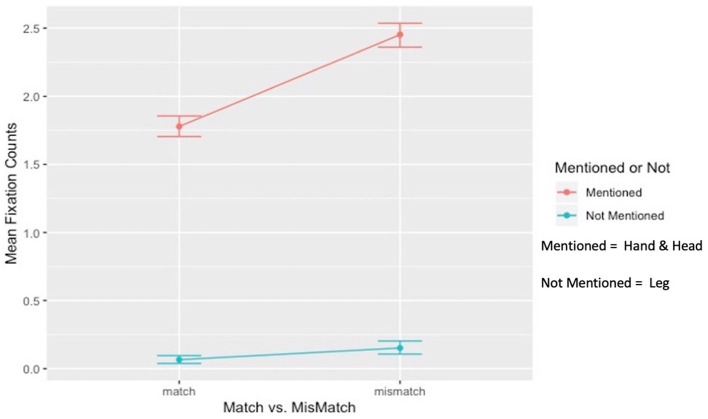
Interaction between mentioned vs. not-mentioned body effector and match vs. mismatch conditions.

The first interaction term examined the effect of mentioned body effectors (Head and Hand) relative to the not-mentioned body effector (Leg) in match and mismatch conditions (Match: *M* = 3.55, *SD* = 1.37; Mismatch: *M* = 4.90, *SD* =1.72; After average, Head + Hand/2; Match: *M* = 1.77, *SD* = 6.85; Mismatch: *M* = 2.45, *SD* = 8.60). The contrast was significant (Figure 6). The effect of the mentioned body effectors compared to the not-mentioned body effector in increasing inspection eye gaze (fixation counts) was significantly stronger in the mismatch condition than in the match condition [*b* = −0.19, *t*(220) = −6.28, *p* < 0.001, *f*^2^ = 0.20]. Moreover, the main effect of mentioned vs. not-mentioned body effector was significant (*b* = 1.33, *SE* = 0.03*, t* = 42.92*, p*<*0.0*01, 95% CI = [1.27, 1.39]), as was the main effect of match-mismatch conditions (*b* = −0.23, *SE* = 0.03*, t* = −8.26*, p* < 0.001, 95% CI = [−0.29, −0.18]). The simple main effect of match vs. mismatch conditions showed that the mentioned Head + Hand had significantly higher fixation counts in the mismatch condition than in the match condition (*t*(335) = 11.30, *p* < 0.001, *d* = 0.87, 95% CI [1.11, 1.58]). Although differences in fixation counts were also found between the match and mismatch conditions for the not-mentioned Leg, the effect size was minimal (d < 0.2); therefore, these differences could be ignored (*t*(335) = −2.97, *p* = 0.05, *d* = 0.17, 95% CI [−0.02, −0.14]). Conversely, the simple main effect of mentioned vs. not-mentioned body effectors showed that fixation counts on the mentioned Head + Hand were significantly higher than on the not-mentioned Leg in both the match (*t*(335) = 44.33, *p* = < 0.001, *d* = 0.35, 95% CI [1.63, 1.78]) and mismatch conditions (*t*(335) = 46.23, *p* = < 0.001, *d* = 0.38, 95% CI [2.20, 2.39]).

#### 3.3.2 Two (Explicitly mentioned: Head vs. Implicitly mentioned: Hand) × Two (Match vs. Mismatch conditions) interaction

**Figure 7:**
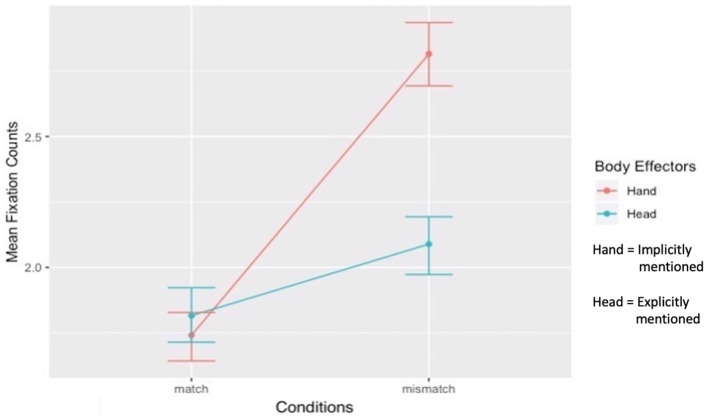
Interactions between explicitly vs. implicitly mentioned body effectors and match vs. mismatch conditions.

The second interaction term examined whether the effect of the explicitly mentioned body effector compared to the implicitly mentioned body effector differed between the match and mismatch conditions. The contrast was significant (Figure 7). The effect of the implicitly mentioned body effector (Hand) compared to the explicitly mentioned body effector (Head) on fixation counts was significantly stronger in the mismatch condition than in the match condition (*b* = 0.20, *t*(220) = 7.41, *p* < 0.001, *f*^2^ = 0.30). The main effect of explicitly vs. implicitly mentioned body effectors was also significant (*b* = −0.16, *SE* = 0.03*, t* = −0.63*, p* < 0.001, 95% CI = [−0.21, −0.10]). The simple main effect of match vs. mismatch conditions showed that fixation counts differed between the match and mismatch conditions for the explicitly mentioned Head (*t*(335) = −3.41, *p* < 0.001, *d* = 0.19, 95% CI [−0.43, −0.11]), though the effect size was minimal and the differences were negligible. Conversely, the implicitly mentioned Hand received significantly higher fixations in the mismatch condition than in the match condition (*t*(335) = −13.24, *p* < 0.001, *d* = 1.09, 95% CI [−1.23, −0.91]). The simple main effect of explicitly vs. implicitly mentioned body effectors revealed that, in the match condition, fixation counts on the explicitly mentioned Head and the implicitly mentioned Hand were comparable (*t*(335) = 1.06, *p* = 0.28, 95% CI [−0.06, 0.21]). Finally, in the mismatch condition, fixation counts on the implicitly mentioned Hand were significantly higher than those on the explicitly mentioned Head (*t*(335) = 8.96, *p* < 0.001, *d* = 0.65, 95% CI [0.56, 0.88]).

#### 3.3.3 Simple main effects of body effectors

We first built a baseline model with only intercept and random effects for participants, estimated using maximum likelihood to analyze the main effect of the body effectors. We then built two additional models: *the match condition model*, which included the match condition as a predictor, and *the mismatch condition model*, which included the mismatch condition as a predictor. The likelihood ratio was computed to compare *the baseline model* with *the match condition model* and *the baseline model* with *the mismatch condition model* [Match: $\chi_{\left(2\right)}^{2}$ = 244.09, *p* < 0.001; Mismatch: $\chi_{\left(2\right)}^{2}$ = 343.75, *p* < 0.001]. Finally, *post-hoc* tests showed that, in the match condition, fixation counts on the Hand and the Head were similar (*z* = 0.94, *p* = 0.61, 95% CI [0.25, −0.10]). Conversely, fixation counts on the Leg in the match condition were significantly fewer than those on the Hand (*z* = −21.35, *p* < 0.001, *f*^2^= 1.28, 95% CI [−1.85, −1.49]) and the Head (*z* = −22.30, *p* < 0.001, *f*^2^ = 1.5, 95% CI [−1.93, −1.56]). In the mismatch condition, fixation counts on the Head were significantly fewer than those on the Hand (*z* = −9.82, *p* < 0.001, *f*^2^ = 0.28, 95% CI [−0.89, −0.55]). Fixation counts on the Leg were also significantly fewer than those on the Hand (*z* = −36.03, *p* < 0.001, *f*^2^ = 3.76, 95% CI [−2.83, −2.49]) and the Head (*z* = −26.20, *p* < 0.001, *f*^2^= 0.81, 95% CI [−2.11, −1.76]).

**Figure 8:**
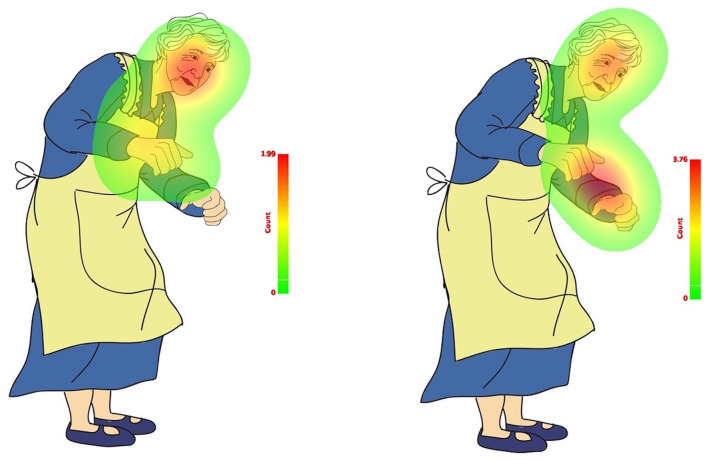
Count-based fixation map of total sample in match (**left**) and mismatch (**right**) conditions.

**Figure 9:**
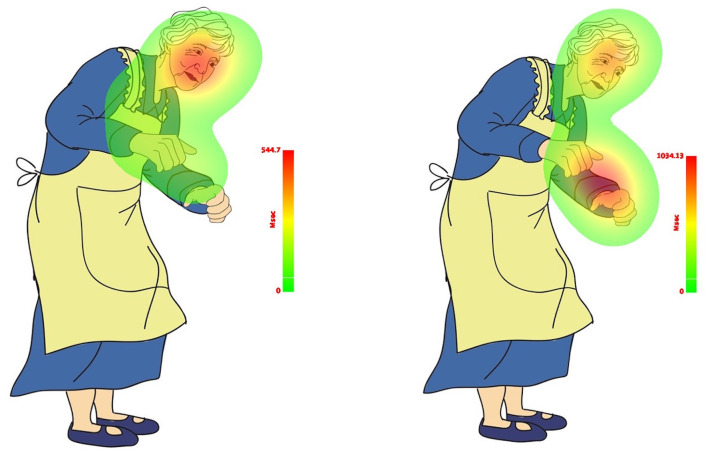
Duration-based fixation map of total sample in match (**left**) and mismatch (**right**) conditions.

The results indicate that the not-mentioned body effector (Leg) did not capture participants' attention as much as the explicitly and implicitly mentioned body effectors (Head and Hands). This trend is illustrated in the count-based fixation and duration-based fixation maps presented in Figures 8, 9. The color legend to the right of the image explains the mapping: warmer colors, such as red, represent higher fixation counts and longer durations.

### 3.4 Results for RQ 2: to what extent are L2 mental representations grounded in motor experiential traces? Specifically, how fast did the participants identify whether the action depicted in the picture matches or mismatches the action implied in the sentence?

To answer RQ 2, we conducted a series of paired t-tests. The analysis revealed no differences between the match and mismatch conditions in the first fixation duration on the Hand (match: *M* = 240.71, *SD* = 151.15; mismatch: *M* = 255.27, *SD* = 117.54, *t*(335) = −1.59, *p* = 0.11, 95% CI [−32.50, 3.39]). Conversely, the first-pass dwell time on the Hand was notably shorter in the match condition than in the mismatch condition (match: *M* = 293.54, *SD* = 157.54; mismatch: *M* = 395.97, *SD* = 198.39, *t*(335) = −7.51, *p* < 0.001, *d* = 0.50, 95% CI [−129.24, −75.60]).

When comparing total dwell time on the Hand between the match and mismatch conditions, it was observed that participants fixated on the Hand significantly longer in the mismatch condition than in the match condition (Match: *M* = 400.51, *SD* = 192.43; Mismatch: *M* = 633.76, *SD* = 310.41, *t*(335) = −11.52, *p* < 0.001, *d* = 0.88, 95% CI [−273.04, −193.44]). Additionally, a square-root transformation of the second-pass dwell time data showed that dwell time was significantly shorter in the match condition than in the mismatch condition (match: *M* = 7.00, *SD* = 7.62; Mismatch: *M* = 14.24, *SD* = 5.92, *t*(335) = −13.28, *p* < 0.001, *d* = 1.15, 95% CI [−8.35, −6.19]). Finally, there was a significant difference between the match and mismatch conditions for the regression-in-count measure. The participants revisited the Hand more frequently in the mismatch condition than in the match condition (match: *M* = 0.50, *SD* = 0.50; Mismatch: *M* = 1.29, *SD* = 0.71, *t*(335) = −16.71, *p* < 0.001, *d* = 1.49, 95% CI [−0.89, −0.70]). Ultimately, this indicates a significant impeding effect in the mismatch condition, primarily evident in late temporal eye movement measures.

## 4 Discussion

This study proposed two research questions to explore motor embodiment in L2 comprehension. The first research question investigated which embodied aspects of mental representations were formed to achieve L2 action sentence comprehension. The second question probed the extent to which these mental representations were grounded in motor experiential traces. Two major findings emerged. First, in the mismatch condition, participants' fixation counts were highest for the implicitly mentioned Hand, followed by the explicitly mentioned Head, and lowest for the Leg. Second, the temporal threshold for identifying the mismatch action was at the first-pass-dwell time. The following discusses how the results were interpreted to answer the two research questions.

### 4.1 Embodied aspects of mental representations are formed for L2 action sentence comprehension

Previous studies on L1 embodiment using the SPVT paradigm have shown that RT facilitation and impediment in the match and mismatch conditions, respectively, indicate that motor–perceptual representations were mentally formed for sentence comprehension (Zwaan and Pecher, 2012; De Koning et al., 2017). Similarly, the present study on L2 using the SPVT found both facilitating and impeding effects. Notably, these effects were also found in global eye movement measures, such as total dwell time on the whole-picture AOI. This indicates that L2 readers form embodied aspects of mental representations for L2 action sentence comprehension before the picture stimuli are presented, as evidenced by their eye gaze when inspecting the picture stimuli.

### 4.2 Eye gaze on picture stimuli revealed motor aspects of mental representations

RQ 1 investigated whether and what motor experiential traces are involved in mental representations formed for L2 action sentence comprehension. To answer this, we examined which parts of the picture stimulus participants focused on and inspected. HP 3 proposed that the match–mismatch conditions of the sentences and the reference status in the sentence of the body effectors would jointly influence participants' eye gaze on the picture stimuli. The results supported HP 3, as the *interaction model* best fits the data on fixation counts. The findings related to fixation counts on each body effector, along with HP 1 and HP 2 for RQ1, are discussed in the following sections.

#### 4.2.1 HP 1: if L2 participants form motor representations for comprehending an action sentence stimulus, their eye gaze will focus on the protagonist's head in the picture stimulus

The mean fixation count on the Leg in match and mismatch conditions is close to zero (< 0.2), indicating that the Leg bears little relation to the implied action and the affordance of the described objects. This finding is expected since the sentence stimuli require inferences related to the hand and arm, and the Leg was neither explicitly nor implicitly mentioned. Conversely, the mean fixation counts on the Hand and Head were above 1 in both conditions. This indicates that the Hand and Head were consistently inspected. Embodied indexing fixation emerges when external objects are mapped to their mental referents (Spivey and Richardson, 2009). Therefore, the participants' focus on the Head in the picture in match and mismatch conditions signals that they were aligning the protagonist in the picture with the mental referent of the protagonist described in the sentence.

HP 1 was confirmed by comparing the mean fixation counts on the Head and Hand. The mean fixation counts on the Head and Hand were comparable in the match condition, indicating that the Head and Hand are equally relevant to the implied action and the affordance of the described objects. On the other hand, the mean fixation counts on the Head in the mismatch condition were comparable with those in the match condition but were significantly fewer than those on the Hand in the mismatch condition. This suggests that the Head was not the body effector executing the action implied in the sentence. However, it played a role in identifying whether the action depicted in the picture matched or mismatched the action implied in the sentence. In this regard, our findings exemplify Ferstl et al.'s (2017) observation that recognizing an action performed by a protagonist is impacted by the protagonist's facial identity.

#### 4.2.2 HP2: if the affordances of the protagonist and the described object are successfully meshed, the eye gaze on the picture will fall on the body effector executing the action implied in the sentence. Moreover, the number of inspections on this body effector will be higher in the mismatch condition than in the match condition

HP2 was supported by the finding that mean fixation counts on the Hand in the mismatch condition were significantly higher than those in the match condition and on the Head in the mismatch condition. This finding is notable because the Head, which signifies the protagonist's facial identity (Ferstl et al., 2017), was explicitly mentioned in the sentence stimuli, whereas the Hand was not. Despite this, fixation counts on the Hand exceeded those on the Head in the mismatch condition. This indicates that the Hand was crucial for determining whether the depicted action matched or mismatched the action implied in the sentence. Moreover, these findings align with Glenberg and Robertson's (1999) IH, indicating that affordances were correctly extracted, inferred, and then successfully meshed. The successful meshing of affordances facilitated the development of motor representations and was crucial for determining whether the action shown in the picture matched or mismatched and could or could not be mapped onto the verbally induced and internally presented motor representations. Notably, the mean fixation counts on the Hand in the mismatch condition were significantly higher than those in the match condition. This implies that participants identified the inconsistency between the depicted action in the picture and the motor representations they had formed based on the implied action.

In summary, comparing the mean fixation counts of the three body effectors revealed that participants formed motor representations of hand actions. Consistent with Glenberg and Robertson (1999), these fixation counts indicated that motor representations were formed by the relationships among body effectors, actions, and the objects affected by those actions. These findings aligned with embodied indexing observed in previous oculomotor studies of L1 embodiment (Lindsay et al., 2013; Speed and Vigliocco, 2014), where language users mapped pictorial elements to the verbally induced mental representations. Importantly, the results of RQ 1 showed that L2 readers formed motor aspects of embodied mental representations to comprehend L2 action sentences. These findings, consistent with Zwaan and Pecher (2012) and Ahn and Jiang (2018), support the idea that L2 reading comprehension is embodied, similar to the results found in L1 studies conducted by Holt and Beilock (2006). Our study, similar to Holt and Beilock's (2006), investigates motor embodiment, but we focus on L2, whereas they focus on L1.

### 4.3 The second earliest temporal indicator marked the temporal point at which the mismatched action was identified

RQ 2 explored to what extent these L2 mental representations are grounded in motor experiential traces. Specifically, HP 4 examined the speed at which participants determine whether the action depicted in a picture matches or mismatches the action implied in a sentence. The results revealed significant differences in fixation on the Hand between the match and mismatch conditions. These differences were found in both early temporal indicators (first-pass dwell time) and late temporal indicators (second-pass dwell time, regression-in-count, and total dwell time). Recall that first-pass dwell time indexes initial processing, with a longer duration indicating the detection of an improbable object (Henderson et al., 1999). Therefore, the significant differences found on the first-pass dwell time indicated that during the first-pass dwell time, participants in the mismatch condition began to notice that the action depicted in the picture mismatched the action implied in the sentence. This raises the question of why these advanced L2 readers detected the mismatch at the second earliest temporal measure (first-pass dwell time) rather than the earliest (first fixation duration).

There are two reasons for this. First, as noted by De Graef et al. (1990), participants initially identified the object (Hand) in the picture, regardless of whether the condition matched or mismatched with the implied action. In other words, without first identifying the depicted limb or hand, participants would not have been able to determine whether the depicted action matched or mismatched the implied action.

Alternatively, the earliest time event rarely yields a significant effect in L2 studies compared to L1 due to processing speed differences between L1 and L2 (Godfroid, 2019). L2 processing speed is generally slower and more arduous than L1. It is well-documented that L2 higher-order and deep-level processing is inefficient and effortful (Francis and Gutiérrez, 2012; Horiba, 1996; Pérez et al., 2019). According to Zwaan and Brown (1996), mental representations in L2 are typically weaker and less detailed than those in L1, often due to fewer inferences being made. This supports the idea that participants in this study developed mental representations with a limited amount of motor experiential traces. In other words, their motor representations were less detailed and less clear regarding the actions implied in the sentences. The clarity of motor representations was sufficient for participants to recognize the action depicted in the picture in the match condition, but it was inadequate in the mismatch condition. This explains why all the late temporal eye-movement indicators, such as total dwell time and regression-in-count, on the Hand in this study, showed significant differences with large effect sizes between the match and mismatch conditions. Participants doubted whether the mismatched action in the picture truly differed from the action implied in the sentence due to the weak and unclear motor representations that they had formed. This uncertainty made them reinspect the conflicting part of the picture more frequently, increasing the regression-in-count and total dwell time on the Hand in the mismatch condition. Therefore, similar to the findings of Vukovic and Shtyrov (2014) and Foroni (2015), which suggest that L2 processing is less embodied in the perceptual aspect, this study indicates that L2 processing is also less embodied in the motor aspect.

Overall, this study shows that advanced L2 readers mentally form sufficient motor embodiment for L2 action sentence comprehension to identify mismatched actions, though not as quickly. Exploring how L2 proficiency differences impact temporal eye movement measures and the strength of motor representations is beyond the scope of this study.

### 4.4 Situated embodied L2 action language comprehension

This study shows that advanced L2 learners mentally form motor representations to comprehend action sentences. These representations include adequate motor experiential traces related to the action implied in the sentence, such as specific hand actions. These findings contradict the conclusions drawn by Pavlenko (2014) and Tian et al. (2020), who argued that L2 comprehension is disembodied. Although both Tian et al. (2020) and this study involved Chinese-speaking learners of English, Tian et al. employed lexical and phrasal stimuli. According to Borghi (2012) and Glenberg and Kaschak (2002), sentence context provides situational clues that guide the implementation of actions and goals. In other words, without sentence contexts, individuals struggle to mesh affordances. The conflicting results between Tian et al. and this study indicate that situational context is crucial for activating motor experiential traces, which promote motor representations formed for L2 action language comprehension. Thus, this study corroborates the findings of Santana and De Vega (2013) and Schuil et al. (2013), highlighting the importance of sentence context in activating motor experiential traces.

### 4.5 From amodal to embodied learning and teaching

Contrary to the amodal symbols (e.g., translation practices) and grammatical structures commonly emphasized in L2 classrooms, recent L2 embodied pedagogy studies focus on improving vocabulary learning through activities such as mime, gestures, and multimodal inputs (Ulbricht, 2020; García-Gámez et al., 2021). While these activities aim to promote bodily experiences of learning, they often overlook the importance of situated context in embodied language comprehension. According to the IH, action should occur within a situated context. Therefore, effective embodied language pedagogy should incorporate activities that engage learners in scenarios requiring specific actions, thereby promoting motor–perceptual experiences relevant and meaningful within the learning context. Since adult L2 learners have already developed a range of situated embodied experiences through their L1, L2 embodied reading pedagogy should aim to connect and enhance these motor–perceptual, experiential traces to L2. For instance, group discussions about visually representing text content can help adult EFL learners with low proficiency tap into their existing motor–perceptual experiences (Shiang, 2018).

## 5 Conclusion and limitations

This study is the first to investigate the motor aspect of mental representations formed during the comprehension of L2 action sentences and to assess how these mental representations are embodied at the sentence level. Compared to previous L2 studies that focused on the lexical level, our findings advance the embodied account of language comprehension by demonstrating that L2 embodied action-language comprehension is context-driven. Overall, this study indicates that L2 is not disembodied. However, some critical item sets contain events with varying levels of daily familiarity, making direct comparisons challenging. For example, “opening a jar of jam” is not as common as “opening a can of coke.” The trade-off between event familiarity and the familiarity of words or phrases for EFL readers, as well as the clarity of the visual presentation of an action, was a factor in this study. Future research could explore how event familiarity impacts readers' responses. Additionally, due to the large and complex nature of eye-tracking data analysis, this study primarily used data from a single task. Future research could investigate the generalizability of these findings to other tasks. This study only included advanced L2 learners; therefore, the results may not apply to L2 learners of other proficiency levels.

## Data Availability

The raw data supporting the conclusions of this article will be made available by the authors, without undue reservation.

## Footnotes

^1^Given that the action sentences in the SPVT were designed to induce mental representations of a text which involves high-level language processing, only proficient L2 readers were recruited.

^2^See Section 2.6 for an explanation.

^3^Also see Section 2.6 for an explanation.

^4^The artist also provided advice on which action can be clearly illustrated and presented in picture.

^5^The excluded items were those containing celebrities as protagonists, some participants inclined to look at the key features of these celebrities (e.g., Kim Kardashian).

^6^The threshold was based on previous studies on perceptual embodiment, such as Ahn and Jiang (2018) with 80%; Chen et al. (2020) with Chinese participants' verification task at 83.05% and comprehension task 70.35%, and English participants' verification task at 86.75% and comprehension task at 74.43%.

^7^As eye-tracking data for L2 learners is usually skewed (Godfroid, 2019), a strict data cleaning approach was employed to minimize the risk of violating statistical assumptions and the complexity of the analyses.

^8^Although Castelhano and Henderson (2008) suggested that viewers can grasp the gist of a visual scene in about 40 ms, the thresholds for fixation count and for RT in this study were chosen based on our examination of the overall raw data.
